# Increasing risk of breakthrough COVID-19 in outbreaks with high attack rates in European long-term care facilities, July to October 2021

**DOI:** 10.2807/1560-7917.ES.2021.26.49.2101070

**Published:** 2021-12-09

**Authors:** Carl Suetens, Pete Kinross, Pilar Gallego Berciano, Virginia Arroyo Nebreda, Eline Hassan, Clémentine Calba, Eugenia Fernandes, Andre Peralta-Santos, Pedro Casaca, Nathalie Shodu, Sara Dequeker, Flora Kontopidou, Lamprini Pappa, Oliver Kacelnik, Anita Wang Børseth, Lois O’Connor, Patricia Garvey, Rasa Liausedienė, Rolanda Valintelienė, Corinna Ernst, Joël Mossong, Mária Štefkovičová, Zuzana Prostináková, Ann Caroline Danielsen, Aikaterini Mougkou, Favelle Lamb, Orlando Cenciarelli, Dominique L. Monnet, Diamantis Plachouras

**Affiliations:** 1 European Centre for Disease Prevention and Control (ECDC), Stockholm, Sweden; 2 Institute of Health Carlos III, Madrid, Spain; 3 Santé publique France, Saint-Maurice, France; 4 Santé publique France, Regional Unit Provence-Alps-French Riviera and Corsica, Marseille, France; 5 Directorate-General of Health, Lisbon, Portugal; 6 Sciensano, Brussels, Belgium; 7 Directorate of Epidemiological Surveillance and Intervention for Infectious Diseases NPHO, Athens, Greece; 8 Norwegian Institute of Public Health, Oslo, Norway; 9 Health Protection Surveillance Centre, Dublin, Ireland; 10 National Public Health Centre under The Ministry of Health, Vilnius, Lithuania; 11 Public Health Technology Center Institute of Hygiene, Vilnius, Lithuania; 12 Health Directorate, Luxembourg, Luxembourg; 13 Regional Public Health Authority, Trenčin, Slovakia; 14 Alexander Dubcek University, Trenčin, Slovakia

**Keywords:** COVID-19, long-term care facilities, outbreaks, vaccines

## Abstract

We collected data from 10 EU/EEA countries on 240 COVID-19 outbreaks occurring from July−October 2021 in long-term care facilities with high vaccination coverage. Among 17,268 residents, 3,832 (22.2%) COVID-19 cases were reported. Median attack rate was 18.9% (country range: 2.8–52.4%), 17.4% of cases were hospitalised, 10.2% died. In fully vaccinated residents, adjusted relative risk for COVID-19 increased with outbreak attack rate. Findings highlight the importance of early outbreak detection and rapid containment through effective infection prevention and control measures.

In 2021, morbidity and mortality among residents in long-term care facilities (LTCFs) dramatically declined with the progressive increase of coronavirus disease (COVID-19) vaccine uptake, still, several outbreaks, including severe cases and deaths, continued to occur during the second part of 2021 [1]. We present data from 240 outbreaks of COVID-19 occurring between 5 July and 3 October 2021, in LTCFs with high vaccination coverage reported by 10 European Union and European Economic Area (EU/EEA) countries in September and October 2021.

## Data collection and analysis

The data collection followed a protocol developed by the European Centre for Disease Prevention and Control (ECDC) to (i) obtain data on COVID-19 outbreaks in LTCFs, (ii) assess the severity of breakthrough infections by vaccination status in LTCF residents and staff, and (iii) assess determinants of infection, hospitalisation and death [2]. Aggregate data were collected through the European surveillance portal for infectious diseases (EpiPulse). Data on COVID-19 outbreaks were reported on a voluntary basis, and included type of LTCF, starting date of the outbreak, ending date of the local vaccination campaign, vaccine product, testing strategy, severe acute respiratory syndrome coronavirus 2 (SARS-CoV-2) sequencing results, and denominator data by vaccination status for residents and staff. Data on COVID-19 cases could either be reported as aggregate data by vaccination status, including the numbers of asymptomatic and symptomatic cases, hospitalised cases and deaths, or as case-based data. COVID-19 was defined according to the EU case definition [3]. We defined fully vaccinated as having completed the primary series of vaccination, with the number of doses according to manufacturers’ recommendations. Data collection in LTCFs was coordinated by ECDC’s national focal points in each country in collaboration with sub-national authorities in charge of outbreak investigations. An outbreak was defined as the occurrence of more than one confirmed COVID-19 case among LTCF residents within a period of 2 weeks, but focal points were also allowed to use national definitions. They were asked to include data on outbreaks irrespective of the number of cases involved.

Pooled attack rates were calculated by country as the sum of COVID-19 cases divided by the sum of residents, with 95% confidence intervals taking into account overdispersion or exact confidence intervals when only one outbreak was reported. The determinants of COVID-19 in LTCF residents were analysed using a mixed-effects generalised linear model with Poisson distribution, with country included as random effect. Adjusted relative risks (RRa) for vaccination against COVID-19 were calculated for fully vaccinated compared to unvaccinated, excluding those partially vaccinated. Confounding and effect modification was assessed for time since completion of vaccination, size and type of LTCF, country, and categories of outbreak attack rate (AR) in residents, calculated from the distribution of the ARs of the 240 outbreaks as AR quartiles with rounded category limits (<7.5%, 7.5 ≤ AR < 20%, 20 ≤ AR < 45%, ≥ 45%). The p value cut-off for statistical significance was set at 0.05 for main effects and at 0.10 for interaction terms. Twenty outbreaks with missing or discordant data for denominators and cases by vaccination status were excluded from the latter analysis.

## Outbreak description and determinants

Data on a total of 240 outbreaks occurring from July onwards were submitted to ECDC between 23 September and 13 October 2021 by 10 EU/EEA countries (Table 1, Supplementary Table S1). Forty-one (17.1%) outbreaks started in July, 146 (60.8%) in August, 51 (21.3%) in September and one (0.4%) in October 2021.

**Table 1:** Descriptive results for residents by country in COVID-19 outbreaks in long-term care facilities, 10 EU/EEA countries, July-October 2021 (n = 17,268 residents)

Country	Outbreaks (n)	Residents (n)	Fully vaccinated (%)	COVID-19 cases (n)	Pooled AR %	95% CI	Median AR (%)	Hospitalised cases		Deaths (n)	CFR (%)
								n	%		
Belgium	20	1,812	96.3	238	13.1	7.9–21.1	11.7	27	11.3	13	5.5
France	34	2,293	94.1	485	21.2	17.2–25.7	18.4	50	10.3	53	10.9
Greece	14	912	88.8	277	30.4	19.2–44.5	33.7	19	6.9	26	9.4
Ireland	9	424	99.8	107	25.2	13.4–42.5	24.1	9	8.4	10	9.3
Lithuania	4	483	91.1	75	15.5	1.8–65.0	37.7	8	10.7	5	6.7
Luxembourg	4	374	96.0	11	2.9	1.4–6.3	2.8	3	–^a^	4	–^a^
Norway	15	301	ND	68	22.6	14.0–34.3	19.4	2	2.9	6	8.8
Portugal	25	1,073	95.8	589	54.9	34.3–74.0	52.4	49	8.3	40	6.8
Slovakia	1	52	96.2	4	7.7	2.1–18.5	7.7	1	–^a^	1	–^a^
Spain	114	9,544	98.9	1,978	20.7	16.4–25.8	14.9	497	25.1	232	11.7
**Total**	**240**	**17,268**	**97.0**	**3,832**	**22.2**	**18.9**–**25.9**	**18.9**	**665**	**17.4**	**390**	**10.2**

AR: attack rate; CFR: case fatality rate; COVID-19: coronavirus disease; EU/EEA: European Union and European Economic Area; ND: no data.

^a^ Percentages not shown when the number of cases is small.

### Residents

Among 17,268 residents, 3,832 (22.2%) COVID-19 cases were reported. The median attack rate (AR) among residents was 18.9% (interquartile range (IQR): 6.7–42.8) and varied between countries, from 2.8% in Luxembourg to 52.4% in Portugal. Overall, of 3,832 COVID-19 cases among residents, 665 (17.4%) were hospitalised and 390 (10.2%) died. Of 2,640 COVID-19 cases from 195 outbreaks in eight countries that provided information on the presence of symptoms, asymptomatic cases accounted for 1,277 (48.4%) of COVID-19 cases in residents.

Information on vaccination status was provided for 16,834 (97.5%) residents by nine countries. Of those, 16,331 (97.0%) were fully vaccinated (country mean: 95.2%; range 88.8−99.8). Residents were vaccinated with only the Comirnaty vaccine (BNT162b2 mRNA, BioNTech-Pfizer, Mainz, Germany/New York, United States (US)) in 97.0% of the LTCFs. The remaining LTCFs reported having used different types of mRNA and/or adenovirus vaccines. The testing strategy for residents was reported for 205 (85.4%) outbreaks. For 181 (88.3%) of those, it was reported that they had tested all residents of the LTCF (n = 146) or all residents in the affected LTCF ward (n = 35).

### Staff

Data on LTCF staff were provided for 220 (91.7%) outbreaks (Table 2). The median AR was 6.5% (IQR: 1.6–14.3). Of 831 cases among staff members, 11 (1.3%) cases were hospitalised and one (0.1%) died. Asymptomatic cases accounted for 39.7% of cases for whom this information was provided (237/597 cases from 195 outbreaks). Information on vaccination status was provided for 10,496 staff members by nine countries. Of those staff members, 9,634 (91.8%) were fully vaccinated (country mean: 83.7%; range 66.7−96.8). LTCF staff members were vaccinated with only the Comirnaty vaccine in 87.8% of the LTCFs. The remaining LTCFs reported having used other vaccines or different combinations of vaccines. The testing strategy for staff members was reported for 184 (76.7%) outbreaks. Of those, for 154 (83.7%), it was reported that they had tested all staff members of the LTCF (n = 117) or in the affected LTCF ward (n = 37).

**Table 2:** Descriptive results for staff members by country in COVID-19 outbreaks in long-term care facilities, 10 EU/EEA countries, July−October 2021 (n = 12,002 staff members)

Country	Outbreaks (n)	Staff members (n)	Fully vaccinated (%)	COVID-19 cases (n)	Pooled AR (%)	95% CI	Median AR (%)	Hospitalised cases		Deaths (n)	CFR (%)
								n	%		
Belgium	19	1,601	87.4	70	4.4	2.6−7.3	4.8	0	0.0	0	0.0
France	16	899	82.8	69	7.7	5.2−11.2	7.0	0	0.0	0	0.0
Greece	14	454	66.7	65	14.3	8.1−24.2	9.5	0	0.0	0	0.0
Ireland	9	628	94.2	45	7.2	3.7−13.5	7.0	0	0.0	0	0.0
Lithuania	4	289	68.9	20	6.9	0.93−8.4	20.3	0	0.0	0	0.0
Luxembourg	4	476	71.7	13	2.7	1.7−4.3	2.9	0	0.0	0	0.0
Norway	15	477	ND	56	11.7	7.7−17.5	10.0	0	0.0	0	0.0
Portugal	24	793	96.3	120	15.1	9.3-23.6	12.9	1	0.8	0	0.0
Slovakia	1	26	88.5	2	7.7	0.9−25.1	7.7	0	0.0	0	0.0
Spain	114	6,359	96.8	371	5.8	4.5−7.5	3.9	10	2.7	1	0.3
**Total**	**220**	**12,002**	**91.8**	**831**	**6.9**	**5.8−8.2 **	**6.5**	**11**	**1.3**	**1**	**0.1**

AR: attack rate; CFR: case fatality rate; COVID-19 coronavirus disease; EU/EEA: European Union and European Economic Area; ND: no data.

### SARS-CoV-2 variants

SARS-CoV-2 sequencing results were available for 95 (39.6%) of 240 outbreaks from the 10 countries. The Delta variant of concern (VOC) was reported in 93 (97.9%) outbreaks, the Alpha VOC and the Gamma VOC each in one outbreak (Supplementary Table S2).

## Determinants of COVID-19 and related hospitalisation and death

The determinants of COVID-19 and of COVID-19-related hospitalisation and death during outbreaks are shown in Tables 3 and 4, respectively. There was a statistically significant interaction between the outbreak AR and being fully vaccinated against COVID-19, for COVID-19 in residents and staff (Table 3) and COVID-19-related hospitalisation, but not COVID-19-related death in residents (Table 4). When pooling RRa that were not statistically different between each other (Q1 and Q2 versus Q 3 and Q 4, Table 3), the RRa for infection when being fully vaccinated in outbreaks with an AR below 20% was 0.42 (95% CI: 0.30−0.62) in residents and 0.27 (95% CI: 0.18-0.40) in staff members, whereas there was no significant association in outbreaks with an AR of 20% or higher. Similarly, for hospitalisation in residents, the pooled RRa for being fully vaccinated in outbreaks with an AR below 45% (Q1, Q2 and Q3 versus Q4, Table 4) was 0.30 (95%: CI 0.18-0.50), whereas there was no significant effect in outbreaks with an AR of 45% or higher.

**Table 3:** Determinants of COVID-19 in long-term facility residents (n = 15,472) and staff members (n = 9,768) during outbreaks in long-term care facilities, 10 EU/EEA countries, July−October 2021

Determinant		Residents			Staff members		
		RRa	95% CI	p value	RRa	95% CI	p value
Outbreak AR in residents							
Q1	AR < 7.5%	Ref		−	Ref		−
Q2	7.5 ≤ AR < 20%	3.04	1.35–6.84	0.007	3.45	1.61–7.39	0.001
Q3	20 ≤ AR < 45%	5.73	2.60–12.60	< 0.001	1.84	0.72–4.68	0.202
Q4	AR ≥ 45%	8.82	4.17–18.66	< 0.001	2.86	1.23–6.65	0.015
Fully vaccinated		0.39	0.19–0.80	0.010	0.33	0.17–0.64	0.001
Interaction outbreak AR in residents x fully vaccinated							
Q2 x fully vaccinated		1.43	0.63–3.28	0.395	0.79	0.35–1.80	0.575
Q3 x fully vaccinated		2.02	0.91–4.50	0.085	3.22	1.22–8.54	0.019
Q4 x fully vaccinated		2.90	1.35–6.22	0.006	5.01	2.08–12.07	< 0.001
Time since end of vaccination campaign in the facility (months)		1.00	0.94–1.06	0.883	1.15	0.99–1.32	0.060
Size of LTCF (number of residents)							
< 40		Ref		−	Ref		−
40 ≤ n < 80		1.00	0.89–1.11	0.956	1.07	0.84–1.35	0.594
≥ 80		1.08	0.97–1.20	0.150	0.87	0.70–1.09	0.235
Type of LTCF							
General nursing home		Ref		−	Ref		−
Mixed general nursing/residential home		1.08	0.97–1.20	0.185	0.99	0.76–1.28	0.926
Residential home		1.02	0.92–1.13	0.731	1.10	0.85–1.42	0.461
Specialised, other, unknown		1.05	0.68–1.64	0.824	0.53	0.07–3.96	0.538

AR: attack rate; CI: confidence interval; COVID-19: coronavirus disease; EU/EEA: European Union and European Economic Area; LTCF: long-term care facility; Q: quartile; Ref: reference value; RRa: adjusted relative risk.

Fully vaccinated: compared with unvaccinated, excluding partially vaccinated (see also Supplementary Table S3).

Mixed-effects generalised linear model with Poisson distribution. Country was included as random effect in the model.

The RRa of the interaction terms indicates the size and level of significance of the difference with the reference, i.e. the smallest outbreak AR category (Q1, first quartile). To calculate the RR for full vaccination vs COVID-19 infection in e.g. Q2 for residents, the RR in Q1 (0.39) should be multiplied by the RR of the interaction of Q2 (1.43), i.e. 0.39 x 1.43 = 0.56 (95% CI: 0.37–0.86). Similarly, the RR for fully vaccinated residents in Q2 vs Q1 is 3.04 x 1.43 = 4.35 (95% CI: 3.62–5.22).

**Table 4:** Determinants of COVID-19-related hospitalisation and death in long-term care facility residents during outbreaks in long-term care facilities, 10 EU/EEA countries, July−October 2021 (n = 16,477 residents)

Determinant		Hospitalisation			Death		
		RRa	95% CI	p value	RRa	95% CI	p value
Outbreak AR in residents							
Q1	AR < 7.5%	Ref		−	Ref		−
Q2	7.5 ≤ AR < 20%	1.43	0.31–6.53	0.644	3.64	2.09–6.34	< 0.001
Q3	20 ≤ AR < 45%	3.73	0.98–14.11	0.053	11.15	6.81–18.24	< 0.001
Q4	AR ≥ 45%	2.26	0.50–10.29	0.282	27.38	16.89–44.38	< 0.001
**Fully vaccinated**		0.19	0.06–0.61	0.005	0.41	0.26–0.64	< 0.001
Interaction outbreak AR in residents x fully vaccinated							
Q2 x fully vaccinated		2.53	0.54–11.96	0.241	−	−	NS
Q3 x fully vaccinated		2.23	0.57–8.71	0.247	−	−	NS
Q4 x fully vaccinated		7.91	1.70–36.90	0.008	−	−	NS
Size of LTCF (number of residents)							
< 40		Ref		−	Ref		−
40 ≤ n < 80		1.31	1.00–1.72	0.046	1.24	0.88–1.75	0.225
≥ 80		1.12	0.87–1.44	0.390	1.39	1.00–1.93	0.047

AR: attack rate; CI: confidence interval; COVID-19: coronavirus disease; EU/EEA: European Union and European Economic Area; LTCF: long-term care facility; Q: quartile; Ref: reference value; RRa: adjusted relative risk.

Mixed-effects generalised linear model with Poisson distribution. Country was included as random effect in the model. Time since the end of vaccination campaign in the facility and type of LTCF were not significantly associated and were removed from the model because of the relatively small number of hospitalisations and deaths in unvaccinated residents (see Supplementary Table S4). The RRa of the interaction terms indicates the size and level of significance of the difference with the reference, i.e. the smallest outbreak AR category (Q1, first quartile). To calculate the RR for full vaccination vs unvaccinated for COVID-19-related hospitalisation in e.g. Q2, the RR in Q1 (0.19) should be multiplied by the RR of the interaction of Q2 (2.53), i.e. 0.19 x 2.53 = 0.47 (95% CI: 0.17–1.30). Similarly, the risk for fully vaccinated residents to be hospitalised in Q2 vs Q1 is 1.43 x 2.53 = 3.62 (95% CI: 2.51–5.22).

### Case-based data

Case-based data were provided for 2,074 COVID-19 cases by 103 LTCFs from Luxembourg (n = 4) and Spain (n = 99). The median age of COVID-19 cases was 86 years (IQR: 80–90) in 1,742 residents and 41 years (IQR: 31–51) in 332 staff members; 59.8% (1,041/1,742) of residents and 76.2% (253/332) of staff members were female. Unvaccinated residents tended to be slightly older than fully vaccinated residents (median age 89 vs 86 years, p = 0.072). Unvaccinated staff members tended to be younger than fully vaccinated staff members (median age 35 vs 43 years; p = 0.079).

Reverse transcriptase-PCR (RT-PCR) cycle thresholds (Ct) values were provided for 201 (9.6%) of the 2,094 cases. The percentage of cases with high Ct values (≥ 30) was almost twice higher in outbreaks with an AR below 20% (51.7%, n = 31/60) than in outbreaks with an AR of 20% or higher (27.0%, n = 38/141) (p = 0.001).

## Discussion

Our study shows that there is a risk for outbreaks of COVID-19 in LTCFs in EU/EEA countries despite very high uptake of the COVID-19 vaccine in LTCF residents (country mean: 95%) and high uptake in staff (country mean: 83.7%). In some cases, the outbreaks had a high AR and resulted in high morbidity and high mortality among residents. The risk for COVID-19-related hospitalisation and death was higher in large LTCFs than in small LTCFs, which may reflect a higher proportion of frail elderly people in large LTCFs or a higher number of contacts with staff and visitors.

Our study also shows that, in fully vaccinated residents and staff, the risk for COVID-19 was much lower compared to unvaccinated residents and staff in outbreaks with an AR below 20%, but not in outbreaks with an AR of 20% or higher, which emphasises the need to rapidly detect and control outbreaks, even in LTCFs with a high vaccine uptake. The increased RR of infection for fully vaccinated residents and staff in outbreaks with a higher attack rate (20% or more) could be due to three underlying mechanisms. First, a high force of infection because of exposure to large viral loads might overcome the immune protection [4]. This could happen during superspreading events (e.g. a highly infectious index case with contacts with many residents) or with repeated exposure in large outbreaks where many residents and staff members are infected. A lower adherence of vaccinated staff and residents to infection prevention control measures such as wearing face masks and practicing hand hygiene, as well as allowing a larger number of visitors, may also contribute to the increased exposure. The observation that Ct values were low in outbreaks with high ARs seems to support a viral dose hypothesis, even though Ct values were only available for a limited number of cases and should be interpreted with caution. Second, this observation could result from differences in frailty in the resident populations in the included LTCFs that may not have been captured by the variables ‘type of LTCF’ and ‘size of LTCF’. Finally, though very unlikely, there is the hypothetical possibility that issues with specific vaccine lots or the vaccine cold chain in LTCFs experiencing large outbreaks might explain this observation. However, none of the participating countries reported any issue that would support this hypothesis. All these hypotheses need to be investigated with appropriately designed vaccine effectiveness studies in LTCFs taking into account additional confounding factors such as underlying comorbidities and frailty of the residents.

Our study has several limitations. First, the reported RRa pertaining to vaccination must be interpreted with caution because of potential selection bias with only a small proportion of residents being unvaccinated. The reasons why these residents were not vaccinated are not known but could be related to factors such as previous infection and/or frailty that may have resulted in fewer contacts and a low AR among unvaccinated residents. A similar bias may have occurred in staff if unvaccinated staff members were more likely to have been previously infected than vaccinated staff members. However, the observed decrease of the effect of vaccination with increasing attack rates is unlikely to have been influenced by this limitation. Second, the hospitalisation data may have been affected by different policies and thresholds for hospitalisation of residents in different countries. Third, results on hospitalisation and death related to COVID-19 in unvaccinated residents should be interpreted with caution because of the low number of events in this category. Fourth, although most countries reported that they were screening all residents and staff in the entire LTCF or in the affected LTCF ward, we cannot exclude that differences in testing strategy may have affected the results. Fifth, the data do not allow to draw conclusions on either the effect of time from vaccination or the effect of the Delta VOC as most of the outbreaks in this study occurred from July to September 2021, which is not a sufficiently long period to show the effect of waning and which coincides with the period when the Delta VOC was predominant [5]. Therefore, the fact that time since the end of the vaccination campaign in early 2021 for most of the LTCF residents, was not significantly associated with the risk of COVID-19 in our study does not exclude that waning immunity played an important role in our findings.

Properly designed studies are needed to assess the effectiveness of COVID-19 vaccines in LTCF residents and the possible role of waning immunity following vaccination in this population. In the meantime, efforts should be made to reach maximum vaccination coverage in LTCF residents and staff. In addition, an additional vaccine dose has been linked to prolonging the immune response and broadening neutralisation against variant strains, including the Delta VOC [6] and studies from Israel and the United Kingdom showed lower rates of confirmed COVID-19 in persons aged 60 and older, or 50 years and older, respectively, after receiving an additional vaccine dose [7,8]. Therefore, an additional dose should be considered for older frail individuals such as LTCF residents [9] and for staff in LTCFs. A recent mapping of vaccination recommendations in the EU/EEA indicated that 30 of 30 countries already started providing a booster dose of vaccine to LTCF residents as at November 2021 [10].

## Conclusions

Our results indicate that, despite high vaccination coverage, COVID-19 outbreaks continue to occur in LTCFs in the EU/EEA. They highlight the importance of early detection and rapid containment of COVID-19 outbreaks in LTCFs, thus to limit broad circulation of SARS-Cov-2, through rapid testing of all residents and staff, timely isolation of cases and ensuring strict infection prevention and control measures including appropriate ventilation, use of face masks, adherence to hand hygiene and the availability of resources to implement these measures. Finally, our findings also emphasise the importance of considering booster doses to protect this vulnerable population.

## Supplementary Data

513000 Supplementary Material

## References

[1] European Centre for Disease Prevention and Control (ECDC). Rapid Risk Assessment: COVID-19 outbreaks in long-term care facilities in the EU/EEA in the context of current vaccination coverage. Stockholm: ECDC; 26 Jul 2021. Available from: https://www.ecdc.europa.eu/en/publications-data/rapid-risk-assessment-covid-19-outbreaks-long-term-care-facilities-eueea

[2] European Centre for Disease prevention and Control (ECDC). Data collection on COVID-19 outbreaks in closed settings with a completed vaccination programme: long-term care facilities, version 2.0. Stockholm: ECDC;3 Sep 2021. Available from: https://www.ecdc.europa.eu/en/publications-data/data-collection-covid-19-outbreaks-closed-settings-completed-vaccination

[3] European Centre for Disease Prevention and Control (ECDC). Case definition for coronavirus disease 2019 (COVID-19), as of 3 December 2020. Stockholm: ECDC; 3 Dec 2020. Available from: https://www.ecdc.europa.eu/en/covid-19/surveillance/case-definition

[4] Kaslow DC. Force of infection: a determinant of vaccine efficacy? NPJ Vaccines. 2021;6(1):51. 10.1038/s41541-021-00316-5 PMC8042006 33846340

[5] Nanduri S Pilishvili T Derado G Soe MM Dollard P Wu H Effectiveness of Pfizer-BioNTech and Moderna Vaccines in Preventing SARS-CoV-2 Infection Among Nursing Home Residents Before and During Widespread Circulation of the SARS-CoV-2 B.1.617.2 (Delta) Variant - National Healthcare Safety Network, March 1-August 1, 2021. MMWR Morb Mortal Wkly Rep. 2021;70(34):1163-6. 10.15585/mmwr.mm7034e3 34437519 PMC8389386

[6] Falsey AR Frenck RW Jr Walsh EE Kitchin N Absalon J Gurtman A SARS-CoV-2 Neutralization with BNT162b2 Vaccine Dose 3. N Engl J Med. 2021;385(17):1627-9. 10.1056/NEJMc2113468 34525276 PMC8461567

[7] Bar-On YM Goldberg Y Mandel M Bodenheimer O Freedman L Kalkstein N Protection of BNT162b2 Vaccine Booster against Covid-19 in Israel. N Engl J Med. 2021;385(15):1393-400. 10.1056/NEJMoa2114255 34525275 PMC8461568

[8] Andrews N, Stowe J, Kirsebom F, Gower C, Ramsay M, Bernal JL. Effectiveness of BNT162b2 (Comirnaty, Pfizer-BioNTech) COVID-19 booster vaccine against covid-19 related symptoms in England: test negative case-control study. Pre-print. medRxiv. 15 Nov 2021. Available from: https://www.medrxiv.org/content/10.1101/2021.11.15.21266341v1

[9] European Centre for Disease Prevention and Control (ECDC). Interim public health considerations for the provision of additional COVID-19 vaccine doses. Stockholm: ECDC;1 Sep 2021. Available from: https://www.ecdc.europa.eu/en/publications-data/covid-19-public-health-considerations-additional-vaccine-doses

[10] European Centre for Disease Prevention and Control (ECDC). Overview of the implementation of COVID-19 vaccination strategies and deployment plans in the EU/EEA. Stockholm: ECDC;11 Nov 2021. Available from: https://www.ecdc.europa.eu/sites/default/files/documents/covid-19-overview-of-the-implementation-of-vaccination-strategies-and-deployment-plans-11-november-2021.pdf

