# Supplementary material for: Increasing risk of breakthrough COVID-19 in outbreaks with high attack rates in European long-term care facilities, July to October 2021
Source: Euro Surveill. 2021 Dec 9;26(49):2101070. doi: 10.2807/1560-7917.ES.2021.26.49.2101070 (PMC8662803; doi:10.2807/1560-7917.ES.2021.26.49.2101070)
Supplement: Supplementary Material [file 21-01070_SUETENS_Supplement.pdf]

This supplementary material is hosted by *Eurosurveillance* as supporting information alongside the article “Increasing risk of breakthrough COVID-19 in outbreaks with high attack rates in European long-term care facilities, July to October 2021” on behalf of the authors who remain responsible for the accuracy and appropriateness of the content. The same standards for ethics, copyright, attributions and permissions as for the article apply. Supplements are not edited by *Eurosurveillance* and the journal is not responsible for the maintenance of any links or email addresses provided therein.

Increasing risk of breakthrough COVID-19 in outbreaks with high attack rates in European long-term care facilities, July−October 2021

Supplement

Table S1. Description of reported outbreaks of COVID-19 in European long-term care facilities (n=240 LTCFs) in 10 EU/EEA countries

	N of LTCFs	%
LTCF type		
General nursing home	160	66.7
Residential home	39	16.3
Mixed LTCF	36	15.0
LTCF for mentally disabled	4	1.7
Other	1	0.4
LTCF size (no of residents)		
<40 residents	71	29.6
40-79 residents	81	33.8
>=80 residents	88	36.7
Index case		
Resident, fully vaccinated	125	52.1
Resident, partially vaccinated	0	0.0
Resident, unvaccinated	9	3.8
LTCF staff member, fully vaccinated	63	26.3
LTCF staff member, partially vaccinated	6	2.5
LTCF staff member, unvaccinated	16	6.7
Other	4	1.7
Unknown	17	7.1
Testing strategy residents		
All residents in LTCF	146	60.8
All residents in affected ward	35	14.6
Symptomatic residents only	9	3.8
Other strategy	15	6.3
Unknown	35	14.6
Testing strategy staff members		
All staff in LTCF	117	48.8
All staff in affected ward	37	15.4
Symptomatic staff only	6	2.5
Other strategy	24	10.0
Unknown	56	23.3
End month vaccination residents		
January 2021	68	28.3
February 2021	106	44.2
March 2021	30	12.5
April 2021	5	2.1
May 2021	1	0.4
>=June 2021	15	6.3
Unknown	15	6.3
End month vaccination staff		

January 2021	63	25.6
February 2021	83	33.7
March 2021	17	6.9
April 2021	2	0.8
May 2021	8	3.3
>=June 2021	14	5.7
Unknown	59	24.0

Table S2. Start month of outbreak and identified SARS-Cov-2 variants of concern (VOCs) reported outbreaks of COVID-19 in European long-term care facilities (n=240) in 10 EU/EEA countries

Month start outbreak	N of LTCFs	%	SARS-Cov-2 VOC			
			B.1.1.7 (Alpha)	B.1.617.2 (Delta)	P.1 (Gamma)	VOC not identified
July 2021	41	17.1	0	20	1	20
August 2021	146	60.8	1	50	0	95
September 2021	51	21.3	0	20	0	31
October 2021	1	0.4	0	1	0	0
Unknown	1	0.4	0	1	0	0

Table S3. Number and percentage of COVID-19 infections in LTCF residents and LTCF staff by vaccination status and outbreak attack rate category in residents

Attack rate category (residents) / vaccination status	Residents			Staff members		
	n/N	% cases	(95% CI)	n/N	% cases	(95% CI)
Quartile 1, AR <7.5%, not stratified	191/6,443	3.0	(2.6-3.4)	70/4,313	1.6	(1.3-2.0)
Fully vaccinated	182/6,291	2.9	(2.5-3.3)	56/3,974	1.4	(1.1-1.8)
Partially vaccinated	0/43	0.0	(0.0-8.2)	2/75	2.7	(0.3-9.3)
Unvaccinated	9/109	8.3	(3.8-15.1)	12/264	4.5	(2.4-7.8)
Quartile 2, 7.5≤AR<20%, not stratified	485/3,953	12.3	(11.3-13.3)	105/2,135	4.9	(4.0-5.9)
Fully vaccinated	452/3,797	11.9	(10.9-13.0)	77/1,935	4.0	(3.2-4.9)
Partially vaccinated	3/12	25.0	(5.5-57.2)	5/29	17.2	(5.8-35.8)
Unvaccinated	30/144	20.8	(14.5-28.4)	23/171	13.5	(8.7-19.5)
Quartile 3, 20≤AR<45%, not stratified	1,140/3,619	31.5	(30.0-33.0)	169/2,023	8.4	(7.2-9.6)
Fully vaccinated	1,097/3,513	31.2	(29.7-32.8)	158/1,915	8.3	(7.1-9.6)
Partially vaccinated	7/13	53.8	(25.1-80.8)	3/14	21.4	(4.7-50.8)
Unvaccinated	36/93	38.7	(28.8-49.4)	8/94	8.5	(3.7-16.1)
Quartile 4, AR ≥45%, not stratified	1,829/2,534	72.2	(70.4-73.9)	331/1,568	21.1	(19.1-23.2)
Fully vaccinated	1,773/2,439	72.7	(70.9-74.5)	314/1,466	21.4	(19.3-23.6)
Partially vaccinated	2/4	50.0	(6.8-93.2)	4/12	33.3	(9.9-65.1)
Unvaccinated	54/91	59.3	(48.5-69.5)	13/90	14.4	(7.9-23.4)

n=number of infections, N=denominator (number of residents or staff in category); 95% CI=95% confidence interval; AR=attack rate in residents

Table S4. Number and percentage of COVID-19-related hospitalisations and deaths in LTCF residents by vaccination status and outbreak attack rate category in residents

Attack rate category (residents) / vaccination status	Hospitalisation			Death		
	n/N	%	(95% CI)	n/N	%	(95% CI)
Quartile 1, AR <7.5%, not stratified	50/6443	0.8	(0.6-1)	19/6443	0.3	(0.2-0.5)
Fully vaccinated	47/6291	0.7	(0.5-1)	18/6291	0.3	(0.2-0.5)
Partially vaccinated	0/43	0.0	(0-8.2)	0/43	0.0	(0-8.2)
Unvaccinated	3/109	2.8	(0.6-7.8)	1/109	0.9	(0-5)
Quartile 2, 7.5≤AR<20%, not stratified	84/3953	2.1	(1.7-2.6)	40/3953	1.0	(0.7-1.4)
Fully vaccinated	79/3797	2.1	(1.7-2.6)	36/3797	0.9	(0.7-1.3)
Partially vaccinated	1/12	8.3	(0.2-38.5)	1/12	8.3	(0.2-38.5)
Unvaccinated	4/144	2.8	(0.8-7)	3/144	2.1	(0.4-6)
Quartile 3, 20≤AR<45%, not stratified	202/3619	5.6	(4.9-6.4)	113/3619	3.1	(2.6-3.7)
Fully vaccinated	192/3513	5.5	(4.7-6.3)	108/3513	3.1	(2.5-3.7)
Partially vaccinated	1/13	7.7	(0.2-36)	0/13	0.0	(0-24.7)
Unvaccinated	9/93	9.7	(4.5-17.6)	5/93	5.4	(1.8-12.1)
Quartile 4, AR ≥45%, not stratified	286/2534	11.3	(10.1-12.6)	183/2534	7.2	(6.2-8.3)
Fully vaccinated	282/2439	11.6	(10.3-12.9)	170/2439	7.0	(6-8.1)
Partially vaccinated	0/4	0.0	(0-60.2)	0/4	0.0	(0-60.2)
Unvaccinated	4/91	4.4	(1.2-10.9)	13/91	14.3	(7.8-23.2)

n=number of COVID-19-related hospitalisations and deaths, respectively, N=denominator (number of residents in category); 95% CI=95% confidence interval; AR=attack rate in residents
